# Exhaled Breath Analysis Using Electronic Nose in Cystic Fibrosis and Primary Ciliary Dyskinesia Patients with Chronic Pulmonary Infections

**DOI:** 10.1371/journal.pone.0115584

**Published:** 2014-12-26

**Authors:** Odin Joensen, Tamara Paff, Eric G. Haarman, Ib M. Skovgaard, Peter Ø. Jensen, Thomas Bjarnsholt, Kim G. Nielsen

**Affiliations:** 1 Department of International Health, Immunology and Microbiology, University of Copenhagen, Copenhagen, Denmark; 2 Department of Pulmonary Diseases, VU University Medical Center Amsterdam, Amsterdam, The Netherlands; 3 Department of Mathematical Sciences at Copenhagen University, Copenhagen, Denmark; 4 Department of Clinical Microbiology, Rigshospitalet, Copenhagen, Denmark; 5 Danish Paediatric Pulmonary Service, Rigshospitalet, Copenhagen, Denmark; National Research Council of Italy, Italy

## Abstract

The current diagnostic work-up and monitoring of pulmonary infections may be perceived as invasive, is time consuming and expensive. In this explorative study, we investigated whether or not a non-invasive exhaled breath analysis using an electronic nose would discriminate between cystic fibrosis (CF) and primary ciliary dyskinesia (PCD) with or without various well characterized chronic pulmonary infections. We recruited 64 patients with CF and 21 with PCD based on known chronic infection status. 21 healthy volunteers served as controls. An electronic nose was employed to analyze exhaled breath samples. Principal component reduction and discriminant analysis were used to construct internally cross-validated receiver operator characteristic (ROC) curves. Breath profiles of CF and PCD patients differed significantly from healthy controls p = 0.001 and p = 0.005, respectively. Profiles of CF patients having a chronic *P. aeruginosa* infection differed significantly from to non-chronically infected CF patients p = 0.044. We confirmed the previously established discriminative power of exhaled breath analysis in separation between healthy subjects and patients with CF or PCD. Furthermore, this method significantly discriminates CF patients suffering from a chronic pulmonary *P. aeruginosa* (PA) infection from CF patients without a chronic pulmonary infection. Further studies are needed for verification and to investigate the role of electronic nose technology in the very early diagnostic workup of pulmonary infections before the establishment of a chronic infection.

## Introduction

Cystic fibrosis (CF) and primary ciliary dyskinesia (PCD) are two distinct inherited disorders that share a common complication of defective mucociliary clearance leading to recurrent and chronic upper and lower respiratory tract infection resulting in deteriorating lung function from early age. CF currently affects approximately 35.000 children and young adults in Europe and the prevalence is nearly the same in North America making it the most common life-limiting inherited disease among people of Caucasian heritage [1]–[3]. PCD is less prevalent and most treatment recommendations and guidelines arise from the extensive experience from the treatment of CF [4], [5].

One of the cornerstones in the management of these patients is lifelong microbiological surveillance, which allows detection and characterization of the individual infection [6]. This makes it possible to initiate early aggressive antibiotic treatment, which can delay the onset of chronic infections [7] and prevent the deterioration of lung function [8]. Currently, the diagnosis of infection in the lower respiratory tract relies upon sputum culture for species identification and to determine antibiotic sensitivity. Obtaining sputum specimens can be difficult in patients with minimal sputum production and especially in children with limited ability to cooperate, necessitating methods such as sputum induction, endo-laryngeal suctioning, or bronchoalveolar lavage. Common procedures such as endo-laryngeal suctioning are perceived as a invasive and unpleasant experience by many of the paediatric patients. The microbiological diagnosis can be delayed if the culture sample is of insufficient quality or if circumstances require more time consuming, invasive and costly methods e.g. bronchoalveolar lavage. The above mentioned factors warrant a novel approach to the monitoring and diagnostic workup of respiratory infections and are the basis for the increasing interest in the field of exhaled breath research with the focus on infectious pathogens [9]–[23].

Volatile organic compounds (VOCs) are produced by many microorganisms as part of their metabolism. The study of VOCs in exhaled breath samples and bacterial cultures with various types of mass spectrometry (MS) has identified a wide array of VOCs that may be suitable for use in diagnosing respiratory infections [9], [10]. Some studies have focused on species specific VOCs e.g. 2-aminoacetophenone or hydrogen cyanide as a biomarker for *P. aeruginosa* [11]–[13], [21], although some of these VOCs have later been reported as non-specific [14], [24]. However, the strategy of combining many VOCs, none of which are unique in themselves, but together form a unique VOC-profile, has been successful in discriminating between patients with and without respiratory tract infection using gas chromatography-mass spectrometry (GC-MS) technology [12], [15] and electronic nose technology [17], [22], [23].

Volatile organic compounds of human origin have also been studied in the search of biomarkers of lung diseases, including CF and PCD. Both diseases have been associated with characteristic variations in the concentrations of exhaled volatile organic as well as inorganic compounds [19]. Studies have shown that it is possible to distinguish CF and PCD patients from healthy subjects on basis of exhaled breath analysis using MS technology [15], [20] and hand-held electrochemical devices [3]. In a recent study Paff *et al.* demonstrated that breath profiles from patients with CF and PCD were significantly different compared to healthy subjects [25]; although the numbers of participants were small.

*Pseudomonas aeruginosa* (PA) is the main pathogen responsible for chronic lung infection in CF patients and contributes to progressive deterioration of lung function, respiratory failure, and ultimately lung transplantation or death. Other Gram negative bacteria such as species of the *Burkholderia cepacia* complex are also a leading cause of morbidity and mortality in CF patients, while emerging Gram negative bacteria such as *Stenotrophomonas maltophilia* and *Achromobacter xyloxidans* are associated with a less significant clinical impact [26]. A fast non-invasive diagnostic method that could help to detect these pulmonary infections at an early stage may enable better antibiotic treatment of these infections and reduce their negative impact on lung function. With this study we take a small step towards realization of this ideal.

The aim of this explorative study was to investigate the difference in breath profiles of patients with and without distinct chronic lung infections using an electronic nose. Additionally, we wanted to investigate if the different Gram negative bacteria have an influence on the breath profile of these chronically infected patients. Finally, we wanted to validate and, if possible, repeat the findings from a previous study [25], where an identical electronic nose was used in a similar setup.

## Methods

### Design and study population

The study was a cross-sectional case-control study in which all participants delivered two breath samples at a single study visit. Patients of all ages were recruited during regular outpatient clinic visits at the Paediatric Pulmonary Service at Rigshospitalet, Copenhagen, Denmark, between May 2013 and September 2013 in a random order. Patients were included solely on the basis of their diagnosis and infection status. The inclusion of chronically infected patients was prioritized with the intention to achieve an even distribution of chronically infected and non-infected patients. Patients with a pulmonary exacerbation were included solely by chance.

Healthy participants were included as controls at the Paediatric Pulmonary Service for Lung Disease and an outpatient orthopaedic clinic at Rigshospitalet, Copenhagen, Denmark. Controls with an active use of tobacco or a history of pulmonary disease, inflammatory disease, metabolic, or genetic disorders were excluded. Additionally, controls with a fever or productive coughing 14 days prior to measurement were excluded. No microbiological cultures were obtained from the controls.

The sample size estimation was based on previous studies using the same electronic nose [17], [25], although the current study should be regarded as an explorative study as none of the prior studies aimed to investigate the differences in breath profiles of patients with and without chronic airway infections using the exact same setup.

The diagnosis of CF was based on clinical symptoms in combination with an abnormal sweat test (Chloride >60 mmol/l) and/or identification of mutations in both CFTR-genes. The diagnosis of PCD was based on a combination of clinical symptoms, abnormal ciliary beat pattern on microscopic evaluation of respiratory epithelial biopsies, and identification of an ultra structural defect in the cilia by electron microscopy.

Chronic *P. aeruginosa* infection was defined by the Copenhagen criteria [27]: persistent presence of PA in microbiological culture samples for at least 6 consecutive months, or less when combined with the presence of two or more PA precipitins. The chronicity of the infection by other pathogens such as *A. xylosoxidans*, *S. maltophilia* and species of the *Burkholderia cepacia* complex was determined according to the same criteria. The microbiological diagnosis was overall based on samples obtained by different methods such as expectorated sputum, endo-laryngeal suctioning and bronchoalveolar lavages. Patients were not classified according to common pathogens such as *Staphylococcus aureus* and *Haemophilus infuenzae*, because of the relatively low clinical impact of *H. infuenzae* and because *S. aureus* often occurred in co-infection with other pathogens [26], [28]. Patients with a chronic co-infection were excluded.

Pulmonary exacerbation was determined by the need to start additional antibiotic therapy and the presence of at least two of the following six criteria: change in sputum volume and/or color; increased coughing; increased lethargy, feeling unwell, or increased need for sleep; decreased appetite or weight loss; decrease in lung function ≥10%; increased shortness of breath or new acquired radiologic changes [29], [30]. The status of pulmonary function was determined by the most recent spirometry measurement of FEV_1_ and FVC. Spirometry was performed according ATS/ERS guidelines by a trained bioanalyst using a Jaeger Masterscreen PFT pro (Carefusion, Hoechberg, Germany) [31].

### Ethical approval

The study was approved by the Regional Scientific Ethics Committee (H-2-2012-120) and written informed consent was obtained from all participants and the legal guardians of participants under 18 years of age.

### Exhaled breath sampling

Breath samples were collected using an experimental setup designed and tested by our collaborating partners [25], where a face mask was connected to a modified spacer (Babyhaler, GlaxoSmithKline, Copenhagen, Denmark) allowing inspiration through a VOC-filter (A1, North Safety Products by Honeywell) and exhalation into the spacer. The electronic nose was connected to the spacer via tubes (Oxygen bubble tube, Intersurgical) and the measurement was performed after participants had inspired VOC-filtered air for 5 minutes by tidal breathing. All participants were instructed to refrain from ingestion of anything except water 2 hours prior to the measurement. Measurement of patients was performed at a minimum of 2 hours after inhalation of any nebulized medication. Two measurements per patient were performed separated by an interval of 5 minutes. All measurements were performed by one experienced operator.

### Electronic nose

A commercially available handheld electronic nose was used (Cyranose 320, Intelligent Optical Systems Inc, CA, USA). The Cyranose 320 is a carbon black polymer based chemical vapour analyzer with a nanocomposite sensor array with 32 sensors. Exposing the sensors to a VOC mixture results in a fully reversible swelling of the sensor polymers that results in a change in electrical resistance. The relative change in resistance for each of the 32 sensors was captured as raw data, which was subsequently used in the data analysis. The baseline resistance of all sensors were checked regularly during the study period.

### Statistical analysis

All statistical analyses were performed with SPSS 20.0 (IBM) using only data from the first measurement in order to retain comparability to previous studies and practicable feasibility of this breath test. Raw data from four aqua sensitive sensors was excluded because of unreliable readings due to the humidity in the exhaled breath. A data reduction by principal component analysis with no rotation was conducted, thus reducing the raw data to 4 principal components and capturing >97% of the variance. Students *t*-test was used to find the most discriminating principal components, which were used in the subsequent discriminant analysis. The linear canonical discriminant analysis was then performed with size-dependant prior probability for both groups. The data was internally cross validated by a bootstrapping procedure to reduce the chance of false discovery [32]. A p-value of 0.05 was considered statistically significant. The probability score from the discriminant analysis was used to construct a receiver operating characteristic (ROC) curve, giving an estimate of the accuracy. The sensitivity and specificity were specified at a cut-off point determined by the Youden index. The two most discriminating principal components were used to construct a biplot visualizing the observations in each analysis. Pearson's chi-squared and a students *t*-test were used to compare participants characteristics.

The intra-session reliability of the breath measurements was assessed by calculating the intra-class correlation coefficient (ICC) for each of the 28 sensors. The coefficient of variation was calculated and paired *t*-tests were performed to assess the agreement between the two measurements.

## Results

**Figure 1:**
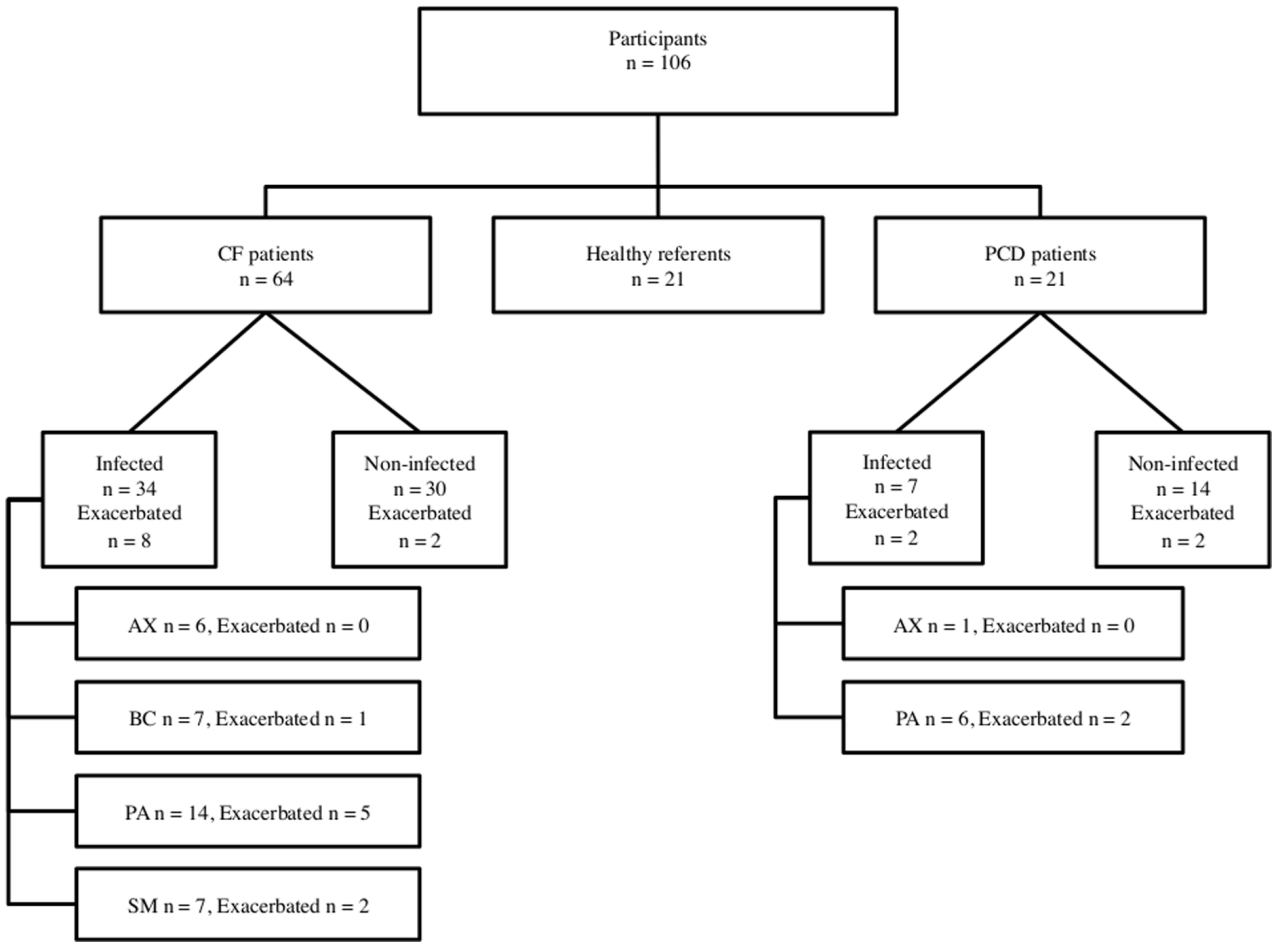
Flowchart displaying the groups of participants and the subgroups of patients according to chronic infection status and exacerbation status. CF – cystic fibrosis. PCD – primary ciliary dyskinesia. AX – *A. xylosoxidans*. BC – *Burkholderia cepacia* complex. PA – *P. aeruginosa*. SM – *S. maltophilia*.

This study included 106 participants, 64 CF patients, 21 PCD patients, and 21 healthy controls whose characteristics are depicted in Table 1 and Fig. 1. No adverse events occurred in association with the sampling procedure. The regular resistance checks and a chronicle depiction of the sensor readings from the whole study period showed no tendency of sensor drift. There was no significant difference in the gender distribution between the three groups and there was no significant difference between the CF and PCD group in lung function parameters, number of exacerbations, or number of chronically infected patients. The mean age in the PCD group was significantly higher than in the CF group (p = 0.020), but comparable to the control group. There was no significant difference in age between the CF group and the control group.

**Table 1:** Patient characteristics.

Characteristic	CF	PCD	Healthy
Subjects (n)	64	21	21
Age (years)^a^^,^ b	17.0 (13.0–25.0)	26.0 (19.0–45.5)	25 (12.0–28.0)
Male (n) (%)	28 (44%)	13 (62%)	8 (38%)
Last FEV1 (% predicted)^a^	86.2 (67.9–95.5)	71.9 (61.9–91.3)	n.a.^d^
Last FVC (% predicted)^a^	99.1 (89.4–112.3)	98.4 (83.1–114.6)	n.a.^d^
Exacerbated (n)	10	4	n.a.^d^
Chronically infected (n)^c^	34	7	n.a.^d^
*P. aeruginosa* (n)	14	6	n.a.^d^
*S. malthophilia* (n)	7	0	n.a.^d^
*A. xyloxidans* (n)	6	1	n.a.^d^
*Burkholderia complex* (n)	7	0	n.a.^d^

a Median (interquartile range).

b Significant difference p<0.05.

c Classification by examination of the previous 6 months of microbiological surveillance.

d Not available.

**Figure 2:**
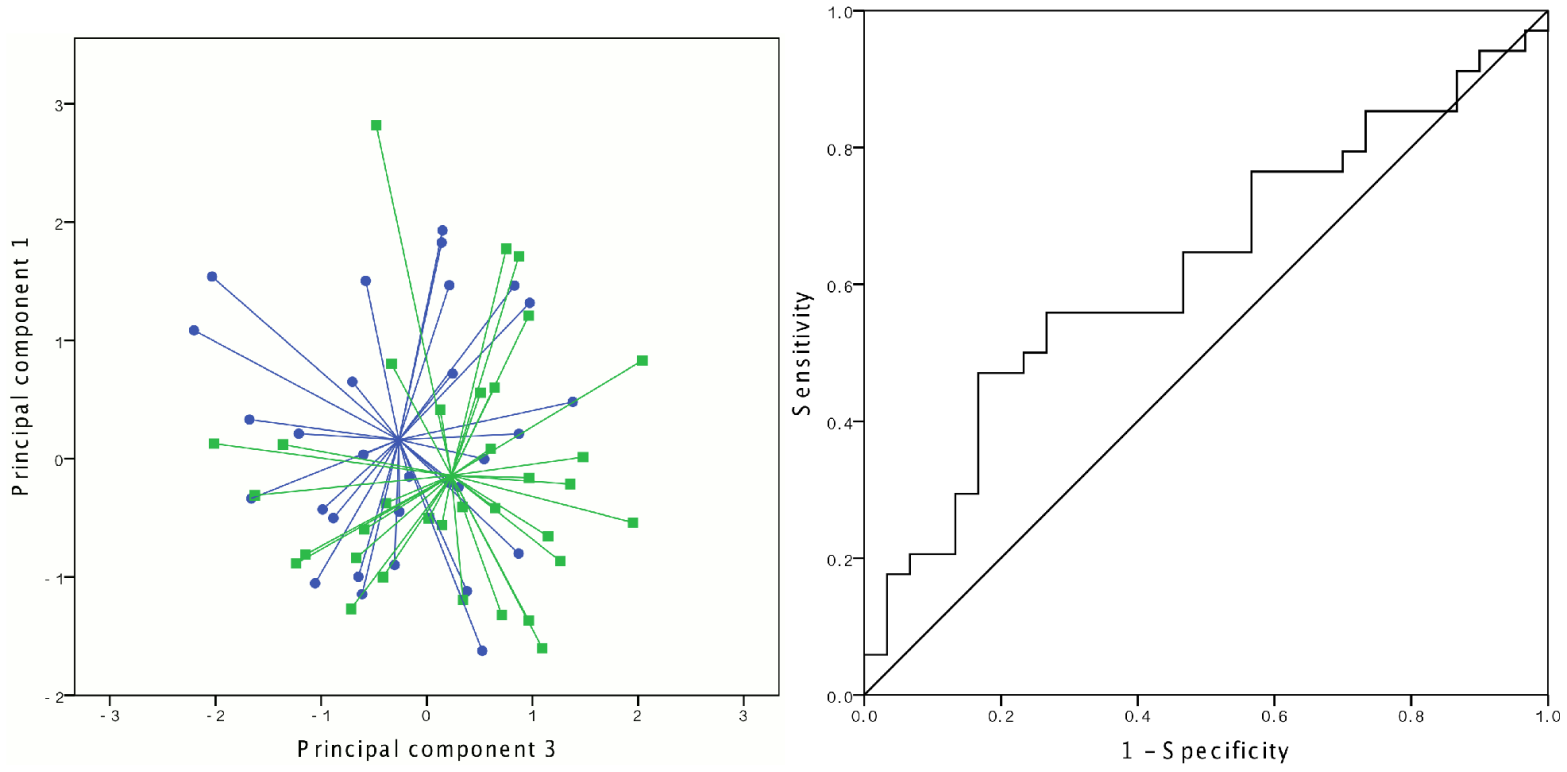
Discrimination of chronically infected vs. non-chronically infected patients with cystic fibrosis. Left: two-dimensional principal component plot visualizing the chronically infected patients with green and non-chronically infected patients with blue. Right: ROC-curve for the discrimination (AUC = 0.59).

**Figure 3:**
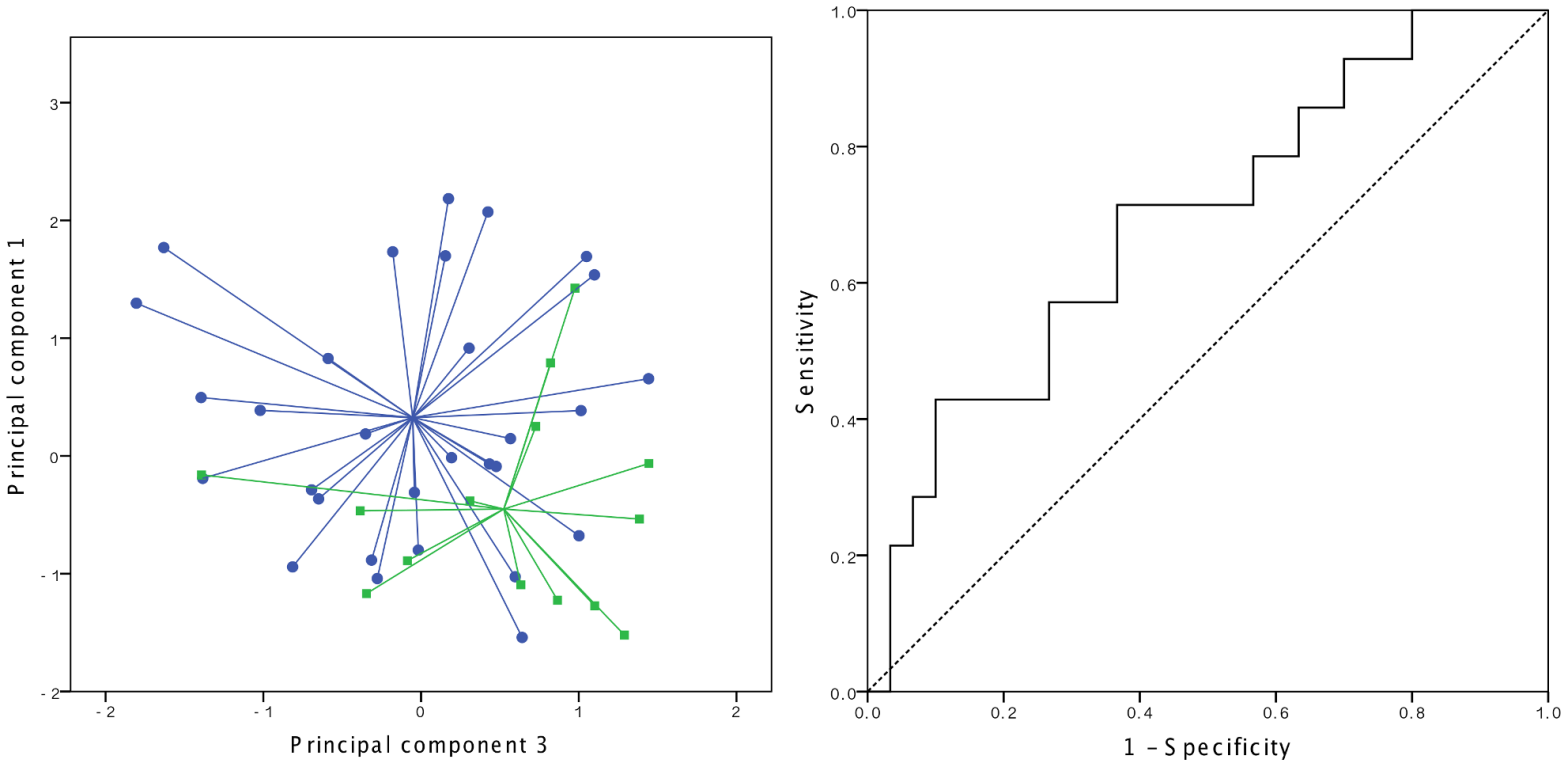
Discrimination of CF patients with a chronic *P. aeruginosa* (PA) infection vs. CF patients without a chronic infection. Left: two-dimensional principal component plot visualizing the PA infected patients in green and the non-chronically infected CF patients in blue. Right: ROC curve for the discrimination of PA infected from the non-chronically infected CF patients (AUC = 0.69).

The breath profiles of CF patients with and without a chronic infection were not significantly different (Fig. 2) and the same analysis of the PCD patients revealed no significantly discriminating principal components. Further analysis revealed a significant difference between the breath profiles of CF patients with a chronic *P. aeruginosa* (PA) infection and CF patients without a chronic infection (p = 0.044) (Fig. 3). The AUC reached 0.69 (95% CI 0.52–0.86) with a sensitivity of 71.4%, and specificity of 63.3%. No significant difference was found between the breath profiles of PCD patients with a chronic PA infection and PCD patients without a chronic infection. No significant difference was observed between the breath profiles of CF patients without a chronic infection and CF patients suffering from chronic infections by *A. xyloxidans*, *Burkholderia cepacia* complex or *S. maltophilia* (results not shown). Furthermore, in a post-hoc analysis no significant difference was found between the breath profile of patients with and without positive cultures sampled at the day of measurement.

The breath profiles of CF patients were significantly different from controls (p = 0.001) as was the case for PCD patients (p = 0.005) and the respective AUC were 0.75 (95% CI 0.64–0.86, sensitivity 50.0% and specificity 95.2%) and 0.75 (95% CI 0.61–0.90, sensitivity 57.1% and specificity 85.7%). In a post-hoc analysis, the exclusion of both chronically infected and exacerbated patients in the analyses had a small negative effect on the discrimination between controls and patients with either disease. No significantly discriminating principal components were found in the comparison of the breath profiles of CF and PCD patients. This finding was unaffected by the exclusion of both chronically infected and exacerbated patients in a post-hoc analysis. The analysis of patients with stable disease and patients with an exacerbation revealed no significantly discriminating principal components in the PCD group, but showed a borderline difference in the CF group (p = 0.057). More details on the results are presented in table 2.

**Table 2:** Results.

Compared groups	N	AUC^a^	95% CI	p-value	Sens. (%)^b^	Spec. (%)^b^
CF Infected vs. non-infected	34 vs. 30	0.59	0.45–0.73	0.206	47.1	76.7
PCD Infected vs. non-infected^c^	7 vs. 14	-	-	-	-	-
CF PA infected vs. non-infected	14 vs. 30	0.69	0.52–0.86	0.044	71.4	63.3
PCD PA infected vs. non-infected^c^	6 vs. 14	-	-	-	-	-
CF vs. Controls	64 vs. 21	0.75	0.64–0.86	0.001	50.0	95.2
- exclusion of infected and exacerbated^d^	28 vs. 21	0.73	0.59–0.87	0.006	64.3	81.0
PCD vs. Controls	21 vs. 21	0.75	0.61–0.90	0.005	57.1	85.7
- exclusion of infected and exacerbated^d^	12 vs. 21	0.75	0.58–0.93	0.017	91.7	47.6
CF vs. PCD^c^	64 vs. 21	-	-	-	-	-
CF exacerbation vs. non-exacerbation	10 vs. 54	0.69	0.55–0.83	0.057	90.0	50.0
PCD exacerbation vs. non-exacerbation^c^	4 vs. 17	-	-	-	-	-

a AUC: Area Under the receiver operating characteristic (ROC) Curve.

b Sens. and spec. are the sensitivity and specificity at the optimum cut-off.

c No significantly discriminating principal components found.

d Post-hoc analysis.

**Figure 4:**
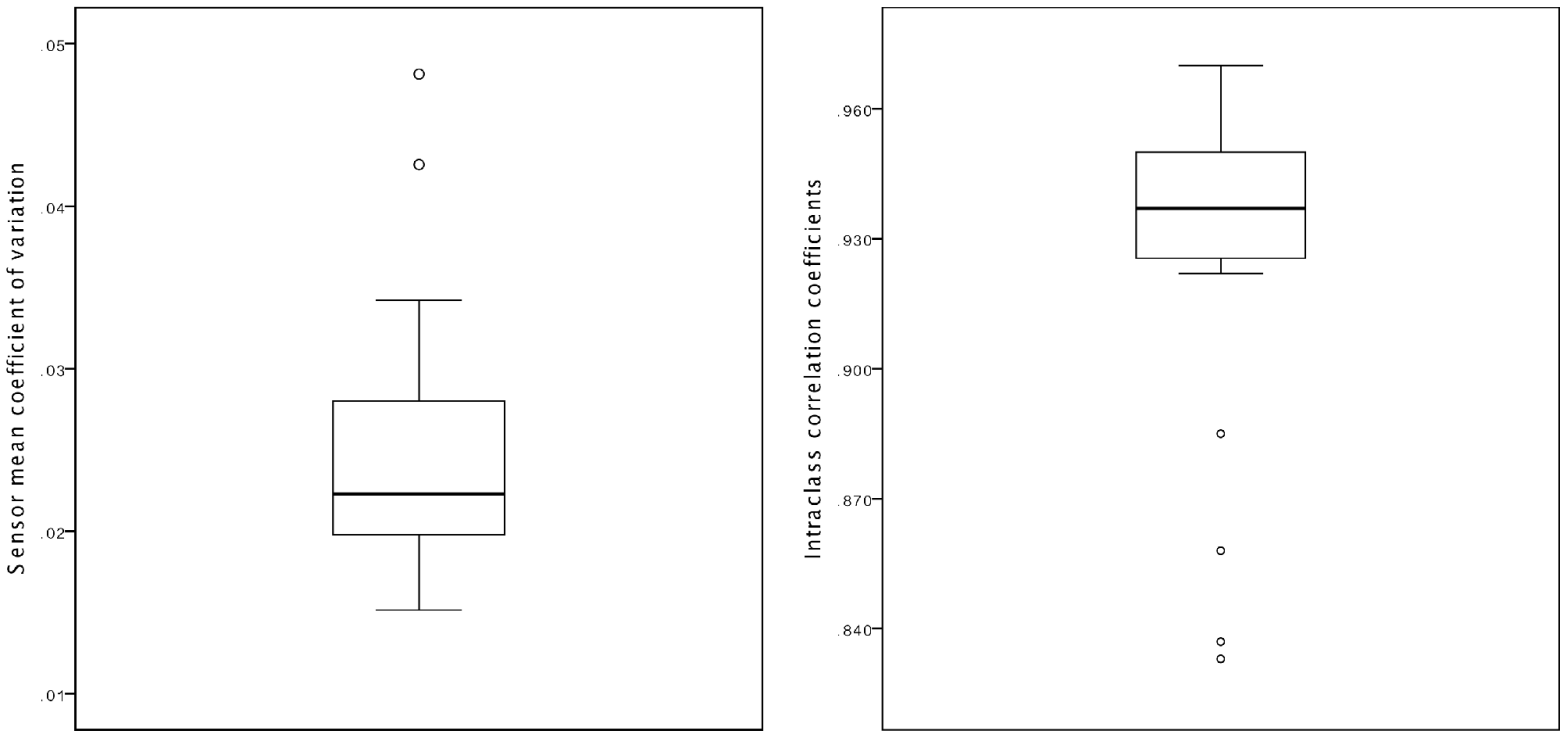
Simple box-plot of the mean coefficient of variation (left) and simple box-plot of the intra-class correlation coefficients (right) of the 28 sensors used in the data analysis.

The mean value intra-class correlation coefficient (ICC) of the 28 sensors was 0.93 (Range 0.83–0.97). The mean coefficients of variation of the 28 sensors were in the range: 0.015–0.048. The paired sample *t*-tests showed that 26 of the 28 sensors had a significantly (p<0.001) higher value in the second measurement compared to the first measurement. An overview of these results are presented in Fig. 4.

## Discussion

The results of this study show that CF patients suffering from a chronic pulmonary *P. aeruginosa* (PA) infection have a significantly different breath profile compared to CF patients without chronic infection. Although, the accuracy of the electronic nose was relatively low AUC = 0.69. These findings support the notion that a chronic pulmonary PA infection may have distinct effect on the exhaled breath profile of CF patients as stated previously in a report by Robroeks *et al*. In this study, CF patients with and without positive *P. aeruginosa* cultures could be discriminated on basis of 14 VOCs from their exhaled breath [15], consequently indicating that the exhaled breath profile driven by the chronic pulmonary PA infection is distinct.

In contrary to expectation, it was not possible to detect any overall difference between chronically and non-chronically infected patients. Additionally, it was also not possible to differentiate non-chronically infected CF patients from CF patients having other chronic pulmonary infections with other pathogens such as: *A. xylosoxidans*, *S. malthophilia* or species of the *Burkholderia cepacia* complex. The number of patients having chronic pulmonary infections with these bacteria and the number of PCD patients with chronic infection was small making it plausible that the study was underpowered to address these comparisons.

The fact that it was possible to discriminate CF patients with a chronic pulmonary PA infection from CF patients without a chronic pulmonary infection may indicate, that these patients have a bacterial specific VOC composition and/or have a different composition of endogenous VOCs in their exhaled breath. *P. aeruginosa* produces and consumes various VOCs that are relatively specific for this species [33] and PA infection in CF patients is associated with a higher pro-inflammatory response in comparison with other pathogens [34], both of which could be contributing factors. Five of the fourteen CF patients with an chronic PA infection suffered from an lung exacerbation, thus lending support to the notion that the inflammatory host response could be a contributing factor. Other factors that could have influenced the result are differences in patient characteristics. Not surprisingly, the CF patients with a chronic pulmonary PA infection were older (p = 0.003), although previous studies have pointed out that age does not directly affect the breath profile [15], [35]. A larger proportion of these patients also suffered from cystic fibrosis related diabetes (CFRD) p = 0.027. Diabetes mellitus (DM), especially type-1 DM, has been associated with variations of different exhaled biomarkers [36], but a study has shown that one of these potential biomarkers, acetone, could not discriminate between 20 healthy controls and 15 CF patients of whom 6 received insulin due to CFRD [20]. CFRD is still a potential confounding factor and its influence needs to be addressed in future studies. Further studies, using appropriate chemical analyses, are needed to verify our findings and to identify the underlying differences in VOC composition in these patients.

The breath profiles of CF patients were significantly different from healthy controls as was the case for PCD patients, confirming the results of the study by Paff *et al* [25]. However, in contrast to this previous study no difference was found between the breath profiles of patients suffering from CF and PCD. Furthermore, no difference was observed between patients with stable disease and patients with an exacerbation in the PCD group, however this difference was borderline significant in the CF group. The number of patients with an exacerbation, especially in the PCD group, was however small, as these patients were recruited solely by chance. A more detailed discussion of the implication of these previously established results can be found elsewhere [25]. Some discrepancy between the current and the previous study was expected, as the previous study was conducted on paediatric patients mainly without a chronic respiratory infection. The patient population in the current study is more heterogeneous in regards to age, relative number of exacerbations, and co-morbidities. Methodological differences e.g. 5 min breath washout and different sterilization procedures could also have had an influence. Additionally, it is currently not possible to manufacture two completely identical units of the Cyranose-320.

The high mean ICC value of 0.93 indicates that the majority of the variance was due to the true variance between the participants and not due to measurement error or the variance within the participants or the sensors. This and the relatively low mean coefficient of variation indicates good intra-session reliability. The paired sample *t*-test showed that 26 of the 28 sensors had a significantly (p<0.001) higher value in the second measurement compared to the first measurement, thus demonstrating a lack of agreement. This indicates that there is a systematic bias towards a higher value in the second measurement. This could be due to a insufficient purging of the sensors after the first measurement, thus making it possible for excess analyte or moisture on the sensors to bias the second measurement. Analyses were performed using only the raw data from the second breath measurement, which resulted in reduced discrimination between the investigated groups. It is therefore important to take appropriate measures to counter this form of bias when designing a study with repeated measurements.

The study was strengthened by the inclusion of a well-characterized population of patients and controls. All patients had complete records with monthly microbiological cultures often dating several years back in time, thus allowing classification according to the criteria mentioned above. Measurements conditions with the electronic nose were standardized in order to minimize the influence of potential confounding factors.

There are two limitations of this study. One is the lack of a gold standard test to determine the presence of a lower respiratory tract pathogen, which is why the infected patients were classified according to the applied clinical criteria also know as the Copenhagen criteria, which are comparable to the Leeds criteria [27], [37]. The infected patients were not classified by the culture result of a sputum sample from the day of the measurement, because evidence suggest that a single sputum sample is inadequate to determine the presence of bacteria in the lower respiratory tract in CF patients [38]. However, we recognize that the applied definition of chronic infection is not ideal, as the classification may be affected by factors such as sample quality variations and the inability of some patients to deliver samples for culture. Disregarding these limitations, the applied definition was the most accurate measure of the presence of pathogenic bacteria available.

A second limitation is that it is not possible to infer anything about the VOC composition of the exhaled breath due to the nature of the applied electronic nose. This makes it difficult to examine the data for confounding factors and sources of bias. A potential biasing factor is the antibiotic therapy, which could explain the lack of discrimination between infected and non-infected, as some studies indicate that the concentration of exhaled inflammatory biomarkers can be affected by antibiotic therapy [20], [39]. CF and PCD patients are subjected to rigorous prophylactic and chronic suppressive antibiotic therapy [40], which could lower the concentration of some discriminating VOCs below the limit of detection of the electronic nose [41]. Differences in treatment in Amsterdam and Copenhagen could also be the cause of some of the discrepancies between the findings by Paff *et al* and our study. Supplementation of the electronic nose measurements with chemical analysis such as GC-MS is therefore highly recommended as such analyses are a necessity when discussing the underlying differences in VOC composition. In addition, the identification of species specific VOCs is essential in the development of novel sensors and the advancement of this field in breath research.

The data presented here show that it is possible to discriminate between CF patients with a chronic pulmonary *P. aeruginosa* infection from CF patients without any chronic pulmonary infection using a non-invasive sampling technique and a multi-purpose commercially available electronic nose. However, since it is definitely of very little clinical value to “detect” a long lasting and established chronic pulmonary infection, this study provides merely a proof of concept. There are several novel techniques emerging such as mid-infrared spectral analysis [42] and nanochips [43], that show great potential as novel biosensors in non-invasive diagnosis of respiratory tract infections. With optimization and standardization of the sampling techniques [44] and employment of such tailored biosensors it will hopefully be possible to detect lower respiratory tract infections at an earlier stage before they are established as chronic infections as defined by traditional methodology and thus increasing the chance of prevention or eradication of infection.

This study adds to the increasing body of evidence that advocates the potential of electronic nose technology in the diagnostic workup of lower respiratory tract infections [17], [22], [23]. However, the relatively low accuracy and the discrepancies of our findings in comparison with the previous study by Paff *et al* demonstrates, that this diagnostic approach requires further refinement before becoming a clinical reality.

## Conclusion

We confirmed the previously established discriminative power of exhaled breath analysis in separation between healthy subjects and patients with CF or PCD. Furthermore, this method significantly discriminates CF patients with a chronic pulmonary *P. aeruginosa* infection from CF patients without any chronic pulmonary infection. Further studies are needed for verification and to investigate the role of electronic nose technology in the very early diagnostic workup of pulmonary infections before the establishment of a chronic infection.

## Supporting Information

S1_Data Minimal data set.
(XLSX)
